# Achieving Textbook Outcomes after Laparoscopic Resection in Posterosuperior Segments of the Liver: The Impact of the Learning Curve

**DOI:** 10.3390/cancers16050930

**Published:** 2024-02-25

**Authors:** Mizelle D’Silva, Jai-Young Cho, Ho-Seong Han, Yoo-Seok Yoon, Hae-Won Lee, Bo-Ram Lee, Mee-Young Kang, Ye-Shong Park, Jin-Ju Kim

**Affiliations:** 1 Department of Surgery, Holy Family Hospital and Research Centre, Bandra, Mumbai 400050, India; mizelledsilva@gmail.com; 2 Department of Surgery, Seoul National University Bundang Hospital, Seoul National University College of Medicine, Seoul 13620, Republic of Korea; hanhs@snubh.org (H.-S.H.); yoonys@snubh.org (Y.-S.Y.); lansh@snubh.org (H.-W.L.); boramlee0827@snubh.org (B.-R.L.); 82637@snubh.org (M.-Y.K.); 82750@snubh.org (J.-J.K.)

**Keywords:** textbook outcomes, laparoscopic liver resection, quality indicators, survival outcomes, posterosuperior segment

## Abstract

**Simple Summary:**

TOs are becoming an important marker not only for assessing hospital and surgeon performance, but also as a predictor of overall survival. As the number of surgeons who achieve the learning curve for laparoscopic resection of tumors in the posterosuperior section of the liver increases, the number of patients with TO will gradually increase with a subsequent improvement in overall survival.

**Abstract:**

Achieving textbook outcomes (TOs) improves the short-term and long-term performance of a hospital. Our objective was to assess TOs in the laparoscopic liver resection (LLR) of tumors in the PS (posterosuperior) section of the liver and identify the impact of the learning curve. We conducted a retrospective cohort study analyzing patients who underwent LLR for lesions located in the PS segments. Patients were divided into a TO and no-TO group. TOs were defined as negative margins, no transfusion, no readmission, no major complications, no 30-day mortality, and a length of stay ≤ 50th percentile. Patients’ outcomes were assessed in two study periods before and after 2015. TOs were achieved in 47.6% (*n* = 117). In multivariable analysis, obesity (*p* = 0.001), shorter operation time (*p* < 0.001), less blood loss (*p* < 0.001), normal albumin (*p* = 0.003), and minor resection (*p* = 0.046) were significantly associated with achieving TOs. Although the 5-year recurrence-free survival rate (*p* = 0.096) was not significantly different, the 5-year overall survival rate was significantly greater in the TO group (*p* = 0.001). Body mass index > 25 kg/m^2^ (*p* = 0.020), age > 65 years (*p* = 0.049), and achievement of TOs (*p* = 0.024) were independently associated with survival. The proportion of patients who achieved a TO was higher after 2015 than before 2015 (52.3% vs. 36.1%; *p* = 0.022). TOs are important markers not only for assessing hospital and surgeon performance but also as predictors of overall survival. As the number of surgeons who achieve the learning curve increases, the number of patients with TOs will gradually increase with a subsequent improvement in overall survival.

## 1. Introduction

Over the past few years, a number of different metrics to measure surgical quality have been described [1]. Here, we evaluated an outcome metric for laparoscopic liver resection (LLR). LLR is considered a low-volume high-risk procedure in most centers. However, at our center with a large surgical volume, LLR represents a high-volume high-risk procedure, and, therefore, outcome metrics are the best choice to evaluate the surgical quality [2]. Textbook outcomes (TOs) are quality indicators of surgical care [3] and have been used to measure the quality of hospitals [4].

The overall survival (OS) of patients with primary liver cancer is gradually improving [5]. However, the incidence of liver cancer is steadily increasing [6,7]. More centers are accepting LLR for primary liver cancer, following the first consensus meeting, which suggested that only tumors located in the anterolateral segments can be safely resected [8]. Nowadays, LLR is being used for donor and recipient hepatectomy [9,10,11]. With technological advances, the importance of the learning curve for different procedures has been established. Laparoscopic resection of the posterior superior segment is considered safe when performed by experienced surgeons [12,13]. LLR in the posterosuperior segments can be selectively performed depending on the size and location of the tumor [14]. The frequency of achieving TOs has become an efficient method for evaluating the effectiveness of a hospital and making comparisons with other expert centers. This metric can also help new centers evaluate their position in the learning curve. Achieving TOs can thus improve the short- and long-term performance status of a hospital. We, therefore, aimed to assess TOs in the LLR of tumors in the posterosuperior section of the liver and identify the impact of the learning curve.

## 2. Materials and Methods

This study was approved by the institutional review board of Seoul National University Bundang Hospital (Approval No. B-2107-696-102). This retrospective study comprised all patients who underwent an LLR of tumors located in the posterosuperior segments of the liver from 2004 to 2020. A total of 246 patients were included in this study.

We evaluated factors associated with achieving TOs, as well as factors associated with survival. We also determined the effect of achieving TOs on long-term survival. In order to account for the learning curve, we additionally assessed these outcomes in two periods: before and after 2015. The inclusion factors included patients with HCC who underwent complete laparoscopic resection of tumors. Patients who were operated on for colorectal liver metastases or intrahepatic cholangiocarcinoma, as well as those in whom laparoscopy was converted to an open procedure, were excluded. 

### 2.1. Definitions

A TO was defined as negative resection margins, no transfusion, no readmission within 30 days, no major complications, no 30-day mortality, and a length of stay (LOS) ≤50th percentile. Patients were divided into two groups according to whether or not a TO was achieved. The posterosuperior segments of the liver include segments 1, 4a, 7, and 8. Complications were graded according to the Clavien–Dindo classification, and complications with a grade ≥3 were classified as severe. The LOS was the time from the first postoperative day to the time of discharge from the hospital. The 50th percentile, i.e., 7 days, was used for the definition of TOs. Major liver resection was defined as resection of three or more consecutive segments of the liver. Thrombocytopenia was defined as a platelet count <100,000/µL. Hypoalbuminemia was defined as a serum albumin of <3.5 mg/dL.

### 2.2. Statistical Analysis

All data were analyzed using SPSS for Windows version 20 (IBM, Chicago, IL, USA). Categorical data are reported as numbers and percentages. Continuous data are expressed as the median and interquartile range. Multivariable regression analysis was carried out to identify factors associated with TOs and survival. All tests were two-tailed, and a *p*-value of <0.05 was considered significant. For the analysis of factors affecting TOs, a *p*-value of <0.1 was considered significant. All predictors with a *p*-value of <0.1 in the univariate analysis were included in the multivariable analysis. OS was calculated as the time from the date of surgery to the date of the last follow-up or death. Recurrence-free survival (RFS) was calculated as the time from the date of surgery to the date of recurrence or the date of the last follow-up, whichever was earlier.

## 3. Results

### 3.1. Patient Characteristics

A total of 246 patients who underwent LLR of tumors located in the posterosuperior segment were included in this study. Minor resection was performed in 196 (79.7%) patients and major resection in 50 (20.3%). Regarding the individual factors used to define TOs, 234 (95.1%) had negative resection margins, 184 (74.8%) did not require transfusion, 243 (98.8%) were alive at 30 days, 235 (95.5%) were not readmitted within 30 days, 208 (84.6%) did not experience major complications, and 144 (58.5%) had an LOS ≤ 50th percentile. TOs were achieved in 117 (47.6%) patients who underwent the LLR of tumors in the posterosuperior segments. TOs were achieved in 52.6% (103/196) of patients who underwent minor resection versus 28% (14/50) of those who underwent major resection, which was significantly different (*p* = 0.002; odds ratio [OR] 0.351, 95% confidence interval [CI] 0.178–0.692). Patients in the TO group were significantly younger (*p* = 0.026). Child—Pugh class was not statistically significant (*p* = 0.057) compared with the non-TO group. The MELD scores did not differ significantly between the two groups. The TO group had a higher proportion of small (<3 cm) tumors (*p* = 0.001) and fewer patients with hypoalbuminemia and thrombocytopenia, which probably contributed to better outcomes in this group. In addition, the operative time (*p* < 0.001) and estimated blood loss (*p* < 0.001) were significantly lower in the TO group. The proportion of patients with anatomical resection was significantly lower in the TO group (Table 1). The multivariable analysis revealed that obesity (*p* = 0.001, OR 2.933, 95% CI 1.516–5.677), shorter operation time (*p* < 0.001, OR 3.870, 95% CI 1.900–7.881), less blood loss (*p* < 0.001, OR 5.663, 95% CI 2.743–11.691), absence of hypoalbuminemia (*p* = 0.003, OR 4.903, 95% CI 1.737–13.843), and minor resection (*p* = 0.046, OR 2.424, 95% CI 1.016–5.785) were significantly associated with the achievement of TOs (Table 2).

### 3.2. Factors Associated with Survival

**Figure 1:**
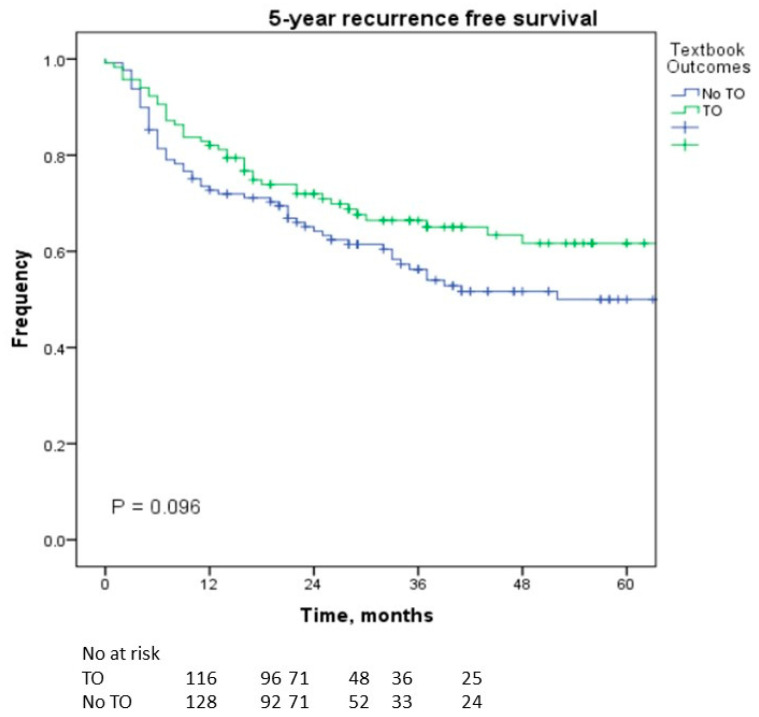
Kaplan—Meier curves of recurrence-free survival according to whether textbook outcomes were achieved.

**Figure 2:**
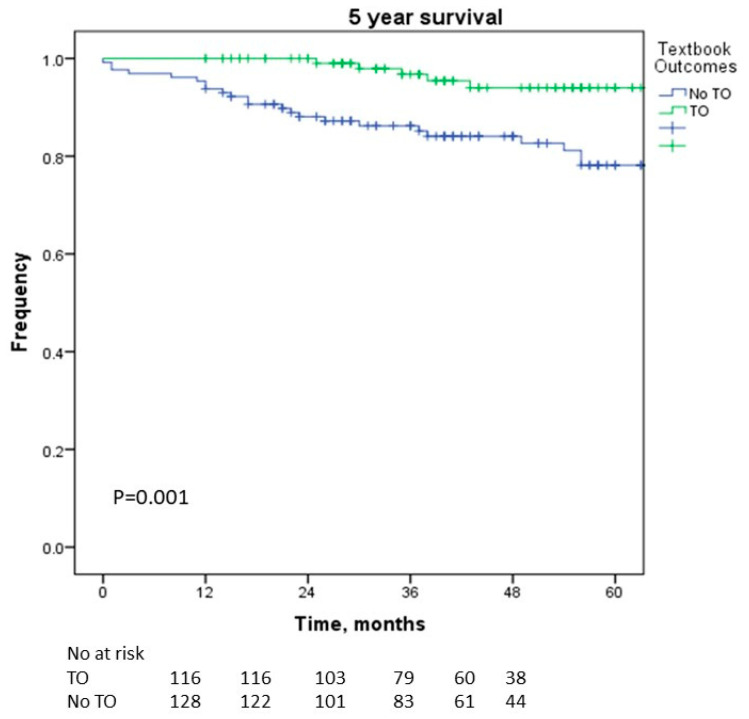
Kaplan—Meier curves of overall survival curves according to whether textbook outcomes were achieved.

In terms of the survival outcomes, we found no significant difference in the 5-year RFS (*p* = 0.096; Figure 1) between the TO and non-TO groups. However, there was a significant difference in the 5-year OS rate between the two groups, and it was greater in the TO group (*p* = 0.001; Figure 2). BMI > 25 kg/mm^2^ (*p* = 0.020, OR = 2.889), age > 65 years (*p* = 0.049, OR = 2.046), and achieving TOs (*p* = 0.024, OR = 3.009) were independently associated with OS in this cohort of patients who underwent LLR in the posterosuperior segments (Table 3).

### 3.3. Learning Curve

**Figure 3:**
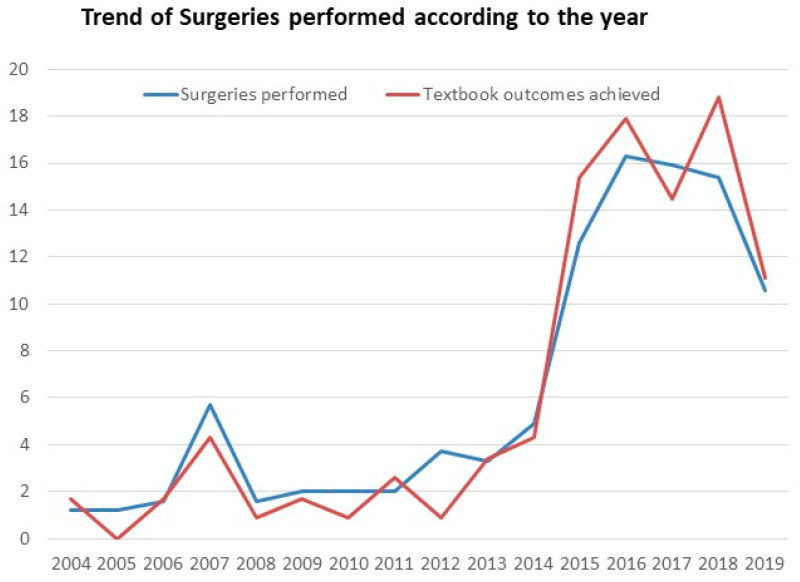
Trends in surgical procedures and the achievement of textbook outcomes over time.

We also examined the trends in surgical procedures over time. The proportion of patients that achieved TOs increased over time with the technological advances and accumulating experience and was significantly greater after 2015 than before 2015 (52.3% vs. 36.1%; *p* = 0.022; Table 4 and Figure 3). When we compared the characteristics of patients who achieved TOs by the year of surgery, we found that the LOS was the most significant factor for the difference in TOs each year. The characteristics of patients who underwent surgery before or after 2015 were compared and shown in Supplementary Table S1. The proportion of older patients was greater (*p* = 0.006), the Pringle maneuver was used more frequently (*n* = 107, 61.5%; *p* < 0.001), and the operation time was significantly shorter (*p* < 0.001) after 2015 compared with before 2015. However, a greater proportion of patients had liver cirrhosis after 2015 (*p* = 0.006). In the multivariable analysis, age > 65 years (*p* = 0.009), operation time (*p* < 0.001), Pringle maneuver (*p* < 0.001), smoking (*p* = 0.010), and liver cirrhosis (*p* = 0.027) were significantly different before versus after 2015 (Supplementary Table S2). 

## 4. Discussion

The resection of tumors in the posterosuperior region is technically challenging, irrespective of the type of surgery, because of the relative posterior and superior location that hinders access to these segments [15]. This region of the liver is located immediately in front of the posterior section of the ribs, adding to the surgical difficulty. These factors limit visibility and make it more difficult to control bleeding [16]. The hand plays an important role in pulling the liver out and performing resection; hence, laparoscopic surgery was generally considered the last procedure in the armamentarium of LLR, even after major surgery.

Laparoscopic techniques were slowly developed to help access the posterosuperior area, such as an implemented transthoracic approach with insertion of an intercostal port [17,18], which was trialed together with changes in the patient’s position and complete retroperitoneal dissection. In addition, the pringle maneuver is being used to reduce blood loss during surgery [19]. With advances in technology and the application of new techniques, it becomes imperative for surgeons to evaluate the outcomes of these procedures. TOs represent one such parameter for evaluating the outcome of surgery, and they are slowly being applied to LLR. Recent publications have assessed the TO for laparoscopic left lateral sectionectomy [20], laparoscopic resection in the anterolateral segment [21], and laparoscopic major liver resection [20]. However, LLR in the posterosuperior section still needed to be evaluated. We achieved TOs in 47.6% of patients who underwent LLR in the posterosuperior segments, similar to the rates achieved in the anterolateral segments and left lateral section. This is notable when we consider the technical difficulty of performing laparoscopic posterosuperior resection. Among the factors included in the definition of TOs, the LOS had the greatest effect on reducing TOs. This is mainly due to the fact that the LOS varies among institutions based on various socioeconomic and cultural factors [22]. TOs were more frequently achieved in patients who underwent minor surgery, further highlighting the difficulty of this procedure, especially when performing major or anatomical resection. Patients with TOs had smaller tumors, more frequently underwent minor resection, and less frequently had hypoalbuminemia or thrombocytopenia, suggesting that their surgery was less demanding, thus increasing the likelihood of achieving TOs. Large tumors are significantly more challenging to resect laparoscopically [13]. Patients with large tumors also tend to have a longer postoperative stay [23]. These factors mean that patients with large tumors are less likely to achieve TOs than patients with small tumors.

We found that the proportion of patients achieving TOs at our center increased significantly after 2015 than before. There was a steep rise in the achievement of TOs after 2015. In addition, after 2015, we were able to perform LLR on a larger number of older patients with a significantly shorter operation time. We also used the Pringle maneuver more frequently after 2015 and the mortality rate was significantly lower. There are several reasons for the rapid increase in TOs after 2015. We started to use the hepatic vein first approach, and intercostal trocars and real-time fluorescence imaging cameras were introduced, and we performed more non-anatomical resections in segments 7 and 8. Moreover, the development of a clinical pathway for liver resection has helped to decrease the LOS. In the early period, LLR in the posterosuperior segments was performed sparingly by surgeons with limited experience and only at advanced centers [24]. However, once the surgeon has gained expertise, the achievement of TOs increases, thus demonstrating the impact of the learning curve. One possible limitation to adopting LLR at new centers is the significant learning curve. The posterosuperior segment was found to be a risk factor for conversion to open surgery, irrespective of the learning curve [18]. Over a few years, a greater number of surgeons would be trained and would slowly overcome their learning curves. The learning curve, as studied previously, varies for each surgical procedure. A recent multicenter study evaluated the learning curve for posterosuperior segments and showed a stepwise improvement in the learning curve beginning with a decrease in the difficulty score followed by an increase and stabilization. They found the learning curve to be 45 cases for wedge resection and 60 cases for anatomical resection [25]. Similarly, another study reported that the learning curve for major laparoscopic resections varied from 45 to 60 cases [26]. Interestingly, the learning curve for LLR was much lower for second-generation surgeons than for pioneer surgeons [27].

We found that the OS was better for patients who achieved TOs than patients without TOs, although there was no difference in RFS. In this cohort of patients who underwent LLR in the posterosuperior segments, the achievement of TOs was independently associated with OS in the multivariable analysis. Similar results were reported for LLR in the anterolateral segments [21]. Thus, achieving TOs is an important factor for improving the OS of patients. Similar results have been reported in other studies [28,29].

This study has a number of limitations. It was a retrospective study spanning a long period of time and comprised a small number of patients. Selection bias may play a significant role in whether patients underwent laparoscopic or open surgery. The LOS is a variable factor, and its inclusion in the definition may further increase the bias. The inclusion of EBL in our analysis may be a confounding factor as blood transfusion was part of the definition of TOs.

## 5. Conclusions

In conclusion, TOs are an important indicator for assessing hospital and surgeon performance and for improving the OS of patients. As the number of surgeons who achieve the learning curve progressively increases, the number of patients with TOs will gradually increase with subsequent improvements in OS.

## Figures and Tables

**Table 1:** Univariate analysis for factors associated with the achievement of textbook outcomes.

	TO ^a^ Achieved (*n* = 117)	TO Not Achieved (*n* = 129)	*p*-Value
Age			0.026
≤65 years	84 (71.8)	75 (58.1)	
>65 years	33 (28.2)	54 (41.9)	
Sex			0.860
Male	90 (76.9)	98 (76)	
Female	27 (23.1)	31 (24)	
BMI ^b^			0.006
<25 kg/m^2^	51 (43.6)	79 (61.2)	
≥25 kg/m^2^	66 (56.4)	50 (38.8)	
Hypertension	53 (45.3)	58 (45)	0.958
Diabetes mellitus	32 (27.4)	36 (27.9)	0.922
Alcohol	50 (42.7)	39 (30.2)	0.042
Smoking	43 (36.8)	31 (24.0)	0.031
Child—Pugh			0.057
A	114 (97.4)	118 (91.5)	
B	3 (2.6)	11 (8.5)	
MELD ^c^	5 (4.3)	11 (8.5)	0.198
Tumor size			0.001
≤3 cm	85 (72.6)	67 (51.9)	
>3 cm	32 (27.4)	62 (48.1)	
T stage			0.013
T1–2	112 (95.7)	111 (86)	
T3–4	5 (4.3)	18 (14)	
Pathological cirrhosis	66 (56.4)	78 (60.5)	0.519
Operation time			<0.001
≤280 min	84 (71.8)	42 (32.6)	
>280 min	33 (28.2)	87 (67.4)	
EBL ^d^			<0.001
≤500 mL	89 (76.1)	47 (36.4)	
>500 mL	28 (23.9)	82 (63.6)	
Thrombocytopenia	7 (6.0)	17 (13.2)	0.064
Hypoalbuminemia	8 (6.8)	23 (17.8)	0.012
Pringle maneuver	61 (52.1)	62 (48.1)	0.523
Type of resection			0.002
Minor	103 (88.0)	93 (72.1)	
Major	14 (12.0)	36 (27.9)	
Anatomical resection	48 (41.0)	80 (62.0)	0.001
Mortality	6 (5.1)	30 (23.3)	<0.001

^a^ TO, textbook outcome; ^b^ BMI, body mass index; ^c^ MELD, model for end-stage liver disease; ^d^ EBL, estimated blood loss.

**Table 2:** Multivariable analysis of factors associated with the achievement of textbook outcomes.

	*p*-Value	OR ^a^	95% CI ^b^
Obesity	0.001	2.933	1.516–5.677
Operation time	<0.001	3.870	1.900–7.881
EBLc	<0.001	5.663	2.743–11.691
Hypoalbuminemia	0.003	4.903	1.737–13.843
Major resection	0.046	2.424	1.016–5.785

^a^ OR, odds ratio; ^b^ CI, confidence interval; ^c^ EBL, estimated blood loss.

**Table 3:** Multivariable analysis of factors associated with overall survival.

	*p*-Value	OR ^a^	95.0% CI ^b^	
			Lower	Upper
BMI ^c^ > 25 kg/m^2^	0.020	2.889	1.182	7.062
Age > 65 years	0.049	2.046	1.004	4.172
TO ^d^ not achieved	0.024	3.009	1.154	7.841

^a^ OR, odds ratio; ^b^ CI, confidence interval; ^c^ BMI, body mass index; ^d^ TO, textbook outcome.

**Table 4:** Factors affecting textbook outcomes according to the year of surgery.

Factor	Before 2015	After 2015	*p*-Value
Transfusion	21 (29.2)	41 (23.6)	0.358
Readmission	3 (4.2)	8 (4.6)	0.882
R1 margins	2 (2.8)	10 (5.7)	0.336
LOS ^a^ > 7 days	43 (59.7)	59 (33.9)	<0.001
Major complication	10 (13.9)	28 (16.1)	0.664
30-day mortality	0	3 (1.7)	0.999

^a^ LOS, length of stay.

## Data Availability

Data are available from the corresponding author upon request.

## Supplementary Materials

The following supporting information can be downloaded at: https://www.mdpi.com/article/10.3390/cancers16050930/s1, Table S1: Univariate analysis of factors according to the year of surgery. Table S2: Multivariable analysis of factors according to the year of surgery.
