# Supplementary material for: Achieving Textbook Outcomes after Laparoscopic Resection in Posterosuperior Segments of the Liver: The Impact of the Learning Curve
Source: Cancers (Basel). 2024 Feb 25;16(5):930. doi: 10.3390/cancers16050930 (PMC10931370; doi:10.3390/cancers16050930)
Supplement: Supplementary file 1 [file cancers-16-00930-s001.zip › cancers-2841931-supplementary.pdf]

Supplementary Table S1: Univariate analysis of factors according to the year of surgery

	Before 2015 (*n* = 72)	After 2015 (*n* = 174)	*P* value
Age			0.006
≤65 years	56 (77.8)	103 (59.2)	
>65 years	16 (22.2)	71 (40.8)	
Sex	51 (70.8)	137 (78.7)	0.186
Male	21 (29.2)	37 (21.3)	
Female			
BMI[b]			0.052
<25 kg/m^2	45 (62.5)	85 (48.9)	
≥25 kg/m^2	27 (37.5)	89 (51.1)	
Hypertension	46 (63.9)	85 (48.9)	0.069
Diabetes mellitus	17 (23.6)	51 (29.3)	0.364
Alcohol	24 (33.3)	65 (37.4)	0.550
Smoking	11 (15.3)	63 (36.2)	0.002
Child–Pugh			0.510
A	69 (95.8)	163 (93.7)	
B	3 (4.2)	11 (6.3)	
MELD[c]	7 (9.7)	9 (5.2)	0.195
Tumor size			0.469
≤3 cm	47 (65.3)	105 (59.2)	
>3 cm	25 (34.7)	69 (39.7)	
Pathological cirrhosis	52 (72.2)	92 (52.9)	0.006
Operation time			<0.001
≤280 min	22 (30.6)	104 (59.8)	
>280 min	50 (69.4)	70 (40.2)	
EBL[d]			0.177
≤500 mL	35 (48.6)	101 (58.0)	
>500 mL	37 (51.4)	73 (42.0)	

Thrombocytopenia	7 (9.7)	17 (9.8)	0.991
Hypoalbuminemia	8 (11.1)	23 (13.2)	0.651
Pringle maneuver	16 (22.2)	107 (61.5)	<0.001
Type of resection			0.243
Minor	54 (75.0)	142 (81.6)	
Major	18 (25.0)	32 (18.4)	
Anatomical resection	43 (59.7)	85 (48.9)	0.122
T stage 3/4	7 (9.7)	16 (9.2)	0.908
Microvascular invasion	30 (41.7)	79 (45.4)	0.592
Satellite nodules	9 (12.5)	12 (6.9)	0.153
Recurrence	47 (65.3)	67 (38.5)	<0.001
Death	22 (30.6)	14 (8.0)	<0.001

[a]TO, textbook outcomes; [b]BMI, body mass index; [c]MELD, model for end stage liver disease; [d]EBL, estimated blood loss.

Supplementary Table S2: Multivariable analysis of factors according to the year of surgery

	P value	OR[a]	95% CI[b]
Age > 65 years	0.009	2.633	1.273–5.448
Operation time	<0.001	3.847	1.962–7.544
Pringle maneuver	<0.001	6.034	3.012–12.086
Smoking	0.010	2.893	1.291–6.484
Liver cirrhosis	0.027	2.193	1.094–4.399

[a]OR, odds ratio; [b]CI, confidence interval.
